# Focused Electron Beam-Based 3D Nanoprinting for Scanning Probe Microscopy: A Review

**DOI:** 10.3390/mi11010048

**Published:** 2019-12-30

**Authors:** Harald Plank, Robert Winkler, Christian H. Schwalb, Johanna Hütner, Jason D. Fowlkes, Philip D. Rack, Ivo Utke, Michael Huth

**Affiliations:** 1 Christian Doppler Laboratory for Direct–Write Fabrication of 3D Nano–Probes (DEFINE), Institute of Electron Microscopy and Nanoanalysis, Graz University of Technology, 8010 Graz, Austria; robertwinkler@felmi-zfe.at; 2 Institute of Electron Microscopy and Nanoanalysis, Graz University of Technology, 8010 Graz, Austria; 3 Graz Centre for Electron Microscopy, 8010 Graz, Austria; 4 GETec Microscopy GmbH, 1220 Vienna, Austria; chris.schwalb@getec-afm.com (C.H.S.); johanna.huetner@getec-afm.com (J.H.); 5 Center for Nanophase Materials Sciences, Oak Ridge National Laboratory, Oak Ridge, TN 37831, USA; fowlkesjd@ornl.gov (J.D.F.); prack@utk.edu (P.D.R.); 6 Materials Science and Engineering, The University of Tennessee, Knoxville, Knoxville, TN 37996, USA; 7 Mechanics of Materials and Nanostructures Laboratory, Empa-Swiss Federal Laboratories for Materials Science and Technology, Feuerwerkerstrasse 39, 3602 Thun, Switzerland; ivo.utke@empa.ch; 8 Physics Institute, Goethe University Frankfurt, 60323 Frankfurt am Main, Germany; michael.huth@physik.uni-frankfurt.de

**Keywords:** scanning probe microscopy, atomic force microscopy, tip fabrication, nano-printing, focused electron beam-induced deposition, 3D printing, nano-fabrication, additive manufacturing

## Abstract

Scanning probe microscopy (SPM) has become an essential surface characterization technique in research and development. By concept, SPM performance crucially depends on the quality of the nano-probe element, in particular, the apex radius. Now, with the development of advanced SPM modes beyond morphology mapping, new challenges have emerged regarding the design, morphology, function, and reliability of nano-probes. To tackle these challenges, versatile fabrication methods for precise nano-fabrication are needed. Aside from well-established technologies for SPM nano-probe fabrication, focused electron beam-induced deposition (FEBID) has become increasingly relevant in recent years, with the demonstration of controlled 3D nanoscale deposition and tailored deposit chemistry. Moreover, FEBID is compatible with practically any given surface morphology. In this review article, we introduce the technology, with a focus on the most relevant demands (shapes, feature size, materials and functionalities, substrate demands, and scalability), discuss the opportunities and challenges, and rationalize how those can be useful for advanced SPM applications. As will be shown, FEBID is an ideal tool for fabrication/modification and rapid prototyping of SPM-tipswith the potential to scale up industrially relevant manufacturing.

## 1. Introduction

Scanning probe microscopy (SPM) techniques, like atomic force microscopy (AFM), scanning tunneling microscopy (STM), or tip-enhanced Raman scattering (TERS), have become an integral part of surface characterization in research and development. During the last decades, a variety of measurement modes have been developed, which go beyond simple morphology assessment to characterizing laterally-resolved surface properties, like electrical conductivity [1], temperature [2], magnetization [3], surface potentials [4], chemical mapping [5], and others [6,7]. Dedicated nano-probes are required to realize these advanced SPM modes, and are just as important as advancements in mechanical design and electronic controls. The proper fabrication of such specialized tips, however, is challenging due to the high demands: tip sharpness for high-resolution imaging, long-term mechanical durability, specific material chemistry for function, and special shapes are only a few issues that have to be considered during fabrication.

Among the various nanofabrication methods, focused electron beam-induced deposition (FEBID) is a promising candidate for tailored manufacturing of SPM tips. In brief, FEBID decomposes surface physisorbed/chemisorbed precursor molecules under continuous electron beam exposure [8,9,10]. Traditionally, FEBID has been used for the fabrication of protective coatings during transmission electron microscopy (TEM) lamella preparation [8,11], photomask repair [12,13,14], circuit editing [8], or for electrically contacting nanowires or carbon nanotubes [15]. During the last 15 years, dedicated FEBID applications have been demonstrated, such as nanolithography [16,17], stress–strain sensors [18], hall sensors [19], gas sensors [20,21], and others [8,22,23,24]. Furthermore, optical applications, such as polarization filters [25], spiral phase plates for vortex beams [26], and photonic–magnetic meta atoms [27] have been demonstrated. In the context of SPM nano-probes, FEBID is well suited to deposit narrow, freestanding nanoscale pillars using the stationary electron beam mode (often denoted as quasi-1D nano-pillar in literature). SPM tips with a high aspect ratio and a tip apex radius of less than 10 nm [28,29,30,31,32] may be routinely achieved using this approach, as discussed in more detail later. In the last five years, 3D-FEBID has experienced a renaissance due to an improved fundamental understanding of the process. 3D-FEBID relies on the controlled lateral movement of the electron beam at slow scan speeds, in the range of nanometer per milliseconds [33]. This mode enables the fabrication of freestanding, inclined nanowires, or segments, acting as fundamental building blocks, which can be combined into complex, mesh-like 3D nanoarchitectures. Recent progress in this 3D nano-printing mode [34] now opens up new pathways for SPM tip fabrication.

This article reviews the literature relevant to SPM tip concepts using 3D-FEBID to demonstrate current capabilities and to trigger further ideas in this technologically challenging field. The article begins with an introduction concerning FEBID and its 3D capabilities, complemented by an overview of boundary conditions for the substrates. The advantages of using FEBID for SPM nano-probe deposition are highlighted together with already demonstrated SPM examples, complemented by a short discussion concerning further opportunities and remaining challenges. For a broader overview of potential 3D-FEBID applications beyond SPM nano-probes, we refer to the literature [34,35].

## 2. The Technology

### 2.1. Focused Electron Beam-Induced Deposition (FEBID)

**Figure 1:**
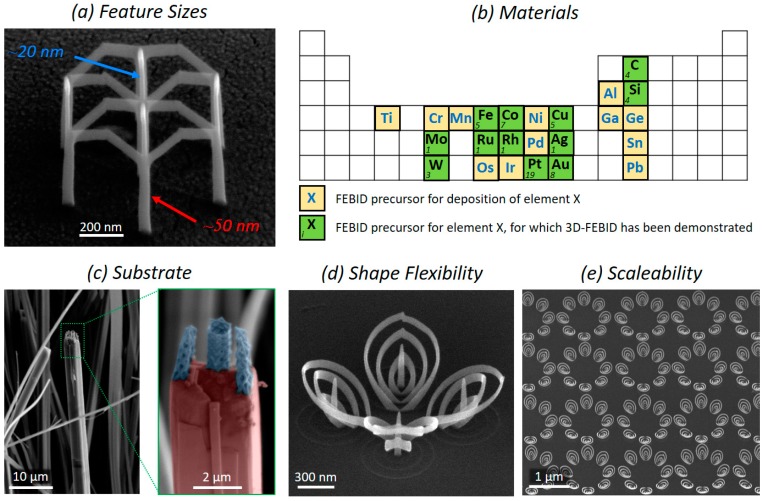
Aspects of 3D nano-printing via focused electron beam-induced deposition (FEBID): (**a**) Feature sizes of a Pt–C multi-pod structure in 52° tilted view. The diameter of individual nanowires is routinely well below 100 nm, while 20 nm can be achieved for special FEBID conditions; (**b**) Materials: a large number of precursors allow the deposition of materials containing different chemical elements [43]. While for some, the fabrication of freestanding 3D objects has been demonstrated (green) in various studies (numbers in the left corners correspond to the number of articles for the respective element) [34], the suitability for 3D-FEBID of other (2D)-FEBID precursors (yellow) is still pending; (**c**) Pt–C based FEBID 3D nano-towers fabricated on top of mineral wires, which is extremely challenging via alternative techniques. Adapted and reprinted from Winkler et al., ACS Appl. Mater. Interfaces 2017 [44]; (**d**) In addition to straight segments, arbitrarily curved nano-wires are possible; (**e**) Fabrication of multiple 3D geometries with 640 elements over several µm² in a single process step.

Image acquisition with a scanning electron microscope (SEM) is accompanied by the formation of a carbon layer in the exposed surface area. The layer forms in response to the electron beam-induced dissociation of hydrocarbon residues ever present in the vacuum chamber of SEMs [36]. These contamination layers are usually unwanted; on the other hand, this localized deposition of material can be used for a controlled direct-write of nanostructures by scanning the electron beam in a preprogrammed way in the presence of a suitably selected adsorbed precursor. This technique, called FEBID, has evolved into a powerful nanofabrication technology. A gas injection nozzle is used to constantly supply precursor molecules for continuous deposition, and depending on the precursor chemistry, other elements beyond carbon can be deposited (see Figure 1b). The nozzle of the gas injection system (GIS) is brought close to the substrate to achieve a locally high precursor surface coverage in the beam impact region, without exceeding the pressure limits of the SEM. In most cases, the gaseous precursor physisorbs, rather than chemisorbs, at the surface, and the molecules diffuse until, after a short residence time, they desorb again, leaving the substrate unaffected. However, in the physisorbed state, the precursor can also be dissociated under electron irradiation by the focused electron beam. This electron-induced decomposition of the precursor results in volatile fragments, which can leave the beam impact region, while non-volatile fragments stick to the surface, forming a solid deposit. By guiding the focused electron beam across the surface, deposits in all dimensionalities, from quasi 0-dimensional (single dots [37]), 1D (lines [38]), 2D (thin films [39]), 2.5D (pads [40]), bulky 3D [41], to vertical pillars [42], are feasible. Beyond that, true 3D-geometries can be attained as discussed in the following, opening up completely new possibilities, thanks to the major progress achieved in recent years [34].

### 2.2. 3D-FEBID

Moving the electron beam in the X/Y plane normally results in deposition on the surface. If the beam scanning speed (also denoted as patterning velocity or writing speed) is reduced to the order of tens of nm per seconds, the deposit can lift off the substrate and form a freestanding nanowire. Furthermore, the nanowire (segment) inclination angle depends on the patterning velocity. Conversely, if the electron beam is kept stationary at one point, a vertical pillar evolves. By that, vertical and tilted nanowires with a typical diameter between 20 and 50 nm can be grown (see Figure 1a). Segment inclination angles ranging from vertical to horizontal are possible, although reliable fabrication in the sub-5° range for segments longer than 1 µm can be challenging. Since position and exposure time (dwell time, DT) of the electron beam in the X/Y plane can be controlled accurately, curved wires (see Figure 1d) and complex multi-branch geometries can be also fabricated. A variety of process parameters (beam, gas, patterning, temperature) influence the 3D growth [33]; the most important are the primary beam energy, beam current, and the patterning velocity. A more detailed discussion will be provided in the respective sections below or can be found in literature [33].

### 2.3. Demands on the Substrate

As mentioned in the previous section, FEBID is a true additive, direct-write 3D fabrication technology, which minimizes the demands on the supporting substrate material and surface topology. The substrate surface need only (1) be vacuum compatible, (2) provide at least low electrical conductivity, (3) withstand electron exposure to a certain degree, and (4) be chemically inert to the precursor material. Demands (1) and (2) can be softened by using an environmental-SEM (ESEM), which allows fabrication in pressure conditions up to about 1 mbar [15], or by thin conductive coatings to prevent electrical charging, respectively. Demand (3) can be mitigated by the application of low-current 3D-FEBID, with the downside of longer fabrication times. Demand (4) is inherently dictated by the precursor chemistry, therefore being invariable. However, for most target elements, alternative precursors are available. Note that many FEBID precursors are non-reactive in combination with most surface materials, although bio-materials are often more sensitive. When considering the SPM cantilever, most material systems are compatible with all four demands, as they are typically fabricated in vacuum conditions anyway. Conveniently, the substrate precursor surface coverage is independent of the surface morphology. Therefore, FEBID can be performed on any substrate topology/geometry which can be accessed by the electron beam [8,45]. For this reason, FEBID supersedes alternative (3D) nano-fabrication techniques that are limited to flat or only slightly curved substrates. Figure 1c shows an example, where 3D-FEBID objects have been placed on a mineral wire, which is extremely challenging to achieve by other nano-fabrication technologies. Although of minor relevance in most cases, we want to mention precursor surface impingement flux aspects in this context. While the adsorption of precursor molecules takes place on any morphology, shadowing effects can occur due to the directional gas flux character from the gas injection nozzle. Substrate diffusion and non-directional precursor adsorption partly compensate for the inhomogeneous surface coverage in the shadow of a morphological barrier [46,47]. Nevertheless, lower deposition rates are not only present behind large obstacles (e.g., higher electrode structures), but also the deposit itself can cause shadowing effects [48,49]. Thus, a proper patterning sequence has to be considered to reduce non-homogeneous growth rate effects during deposition [40]. Also, edges, as typically present when placing a 3D-FEBID tip at the end of a cantilever, result in different deposition rates compared to a flat substrate. Unfortunately, topography variations and/or shadowing can degrade predictability in the 3D growth process. The influence of the precursor surface coverage can be relaxed by operating in the so-called electron limited working regime, where sufficient precursor surface coverage is maximized and constant at a given position, thus lessening the influence of local coverage variations [33]. Often, such working conditions are difficult to establish and an optimization of the GIS alignment and/or design of the GIS are needed.

## 3. Feature Sizes

### 3.1. Intrinsic FEBID Feature Shapes

As discussed in the “Technology” section, the focused electron beam drives precursor dissociation at the surface. The focused electron beam diameter therefore plays a significant role in dictating the spatial resolution of the final deposit. Most SEMs provide beam diameters down to a few nanometers full-width half-maximum (FWHM). Consequently, this should theoretically allow for the fabrication of feature diameters in the same range. However, localized nano-synthesis is most efficient via secondary electrons [50], which are generated not only by primary but also by scattered electrons near the surface. Hence, backscattered electrons (BSE) and forward-scattered electrons (FSE) are involved as well, which expand the spatial range of potential dissociation processes (see [51,52,53,54,55,56] for detailed simulations of FEBID growth). This explains why real feature sizes are about an order of magnitude larger than the primary beam diameter. The exactly achievable dimensions vary with the primary beam parameters and the precursor material, both impacting the electron mean free paths and, by that, the statistical interaction volume in solid materials. To give concrete numbers for nanowires produced by the often used Pt-based Me_3_CpMePt(IV) precursor (see Figure 1b), feature dimensions mostly range between 20 nm and 60 nm in 3D space, but can go below 5 nm under special conditions [8,57,58]. With such small feature sizes, 3D-FEBID exceeds the achievable resolution of other 3D printing techniques [10]. Together with the low demands on the substrate and the high architectural flexibility, 3D-FEBID fulfills the high demands for controlled SPM tip fabrication from a morphological point of view.

### 3.2. SPM Relevant Tailoring

**Figure 2:**
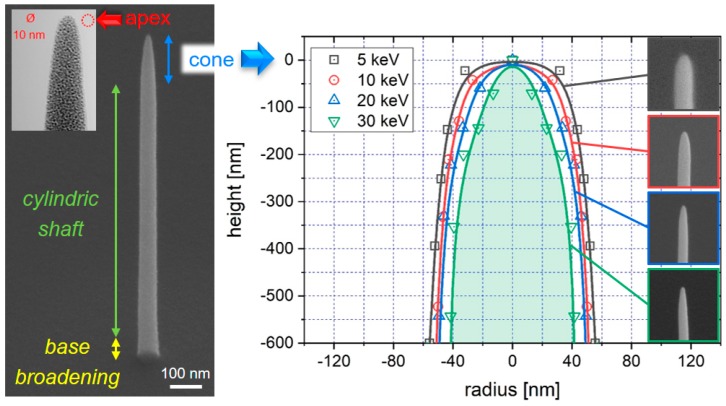
FEBID nano-pillar characteristics. The tilted scanning electron microscope (SEM) image at the left shows the typical nano-pillar morphology, which can be categorized into three vertical sections: a slightly broader base section close to the substrate, a cylindrical shaft region, and the topmost cone, terminated by the apex. The latter can exhibit tip radii down to 5 nm, as shown by the transmission electron microscopy (TEM) inset top left. While the shaft length is proportional to the growth time, the vertical expansion of the conical region scales with the primary electron energy due to the varying mean free paths of electrons, statistically described by the interaction volume. The vertical expansion of the latter decreases with lower primary energies, which explains the higher curvature of the tip region (compare 5 keV/squares with 30 keV/inverted triangles). The insets show representative SEM images of the tip region in a titled view.

The lateral resolution achievable in SPM-based methods is conceptually related to the tip radius at the apex [59]. The first studies go back to the early 1990s, where Hübner et al. [60] and Schiffmann et al. [61] studied the relationship between tip shapes (diameter, height, and conical angle) and fabrication parameters, simply using carbon contaminants inside the SEM, and delivered one of the first proof-of-principles for FEBID-based AFM tip modification. Meanwhile, the basic understanding of the process has strongly improved, which allows specific tailoring. For 3D-FEBID structures, the achievable tip resolution depends on several process parameters. Figure 2 shows a tilted SEM image of a nano-pillar, deposited using Me_2_Au(acac) as the precursor. The overall shape of a pillar can be divided into three distinct regions, as indicated in Figure 2: (1) the cylindrical shaft with high parallelism (Δα < 2°) if fabricated properly, (2) the topmost conical region, and (3) the apex region [8]. In many cases, a small narrowing at the base occurs (see lowest parts in Figure 2), which appears for medium and high beam currents. This morphological peculiarity is the result of an enhanced lateral deposition rate due to the stronger precursor diffusion from the large substrate reservoir and pillar diameters, which varies over a length scale comparable to the precursor-specific average diffusion length (~150 nm for Me_3_CpMePt(IV) [48,51,62]). Such widening can be advantageous for SPM applications due to the improved bonding as a consequence of the larger interface area. The length of the shaft can be precisely adjusted by the growth time, which allows for height-to-width aspect ratios higher than 100. The vertical dimension of the topmost conical region scales with the primary energy [63], as it directly mimics the upper part of the electron interaction volume in the deposit material, which statistically forms by the energy dependent mean free paths of electrons in matter/scattering events. The right graph in Figure 2 gives a direct comparison of the topmost pillar region, where the sharper conical shape for higher primary electron energies is evident. As mentioned at the beginning, sub-10 nm apex radii can be routinely achieved via FEBID for low beam currents (<100 pA) without any further treatments, as exemplarily shown by the transmission electron microscopy (TEM) micrograph inset top left.

**Figure 3:**
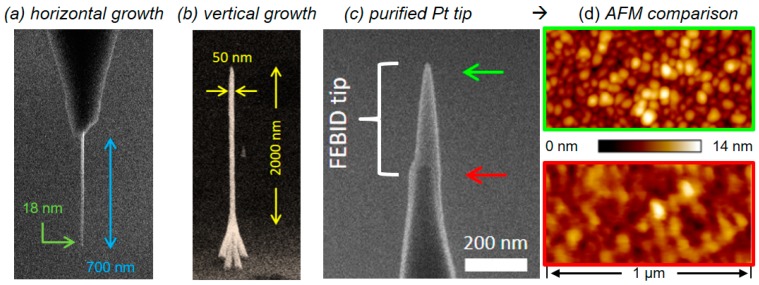
Feature sizes, 3D shape flexibility, and scanning probe microscopy (SPM) advantages: (**a**) shows a horizontally grown Pt–C FEBID tip, where the beam moves across the original tip edge (top-down in this image) with scan speeds around 30 nm/s. The resulting nanowires reveal constant widths in the sub-20 nm regime along the entire tip; (**b**) vertically grown 3D tip, composed of a four-legged base, which then converges to a tall single pillar. While the latter enables high aspect ratio measurements, the former increases the mechanical stability and the adhesion to the surface; (**c**) shows a FEBID modification (green arrow) of a Pt–Ir coated conductive-atomic force microscopy (C-AFM) tip (red arrow). The tip was also fully purified, revealing a tip radius below 10 nm, which strongly improves the lateral resolution capabilities, as shown in (**d**) by a direct comparison of FEBID (top) and the original Pt–Ir tip (bottom), performed on a nanogranular gold sample. The scan width is 1 µm, while both Z scales span across 14 nm, as indicated.

In principle, FEBID-based 3D tip deposition can be performed in two different configurations. In one configuration, the tip is mounted vertically in the SEM (parallel to the electron beam axis) and the electron beam is held stationary; the deposit grows vertically along the electron beam axis. In the alternative configuration, the tip is mounted sideways in the SEM and the electron beam is scanned along the AFM tip axis; the deposit grows laterally. In either configuration, the resulting deposit orientation with respect to the AFM tip is the same, but the resolution is different. The latter, or lateral scanning approach, is more favorable to achieve the smallest tip widths. Such a tip is shown in Figure 3, with a length of 700 nm and an almost constant width of 18 nm. The minimum resolution is achieved using the highest possible primary electron energy on the SEMs (mostly 30 keV), which reduces the elastic scattering probability during beam transmission through the thin nanowire. Consequently, the number of BSE, FSE, and related secondary electrons (SE-II and SE-III, respectively) emitted from the surface are reduced and the beam diameter alone, broadened by the secondary electron (SE-I) action radius, determines the deposit width. The thickness of the growing wire, parallel to the incoming electron beam, can be controlled by the patterning velocity and, in the ideal case, approaches the same values as for the width [29]. Tips deposited in this way are ideal for pinhole characterization or imaging of high aspect ratio side walls. The problem with such tips, however, is their fragile character due to their small cross-sectional area and a comparable small interface area at the pillar–tip interface. The vertical fabrication configuration can be of advantage under these circumstances. For example, while a single pillar fabricated by stationary beam exposure (Figure 2) still has a small interface area, 3D-FEBID can be used as shown in Figure 3b to overcome this fragility problem. The four-legged structure (Figure 3b) deposited by 3D-FEBID provides a more stable base, while still converging to the required vertical single pillar. While the single pillar is well-connected to the merging branches of the tetrapod, the FEBID/sample interface area is four-times higher than for single pillars. Ideal pillar heights are below 1 µm to minimize lateral flexing for reliable AFM operation. Compared to laterally grown tips (see Figure 3a), vertical 3D tips have larger tip diameters of ~50 nm but are radially symmetrical, while apex radii are typically below 10 nm (Figure 2). As mentioned before, the main problem with both tip types is the lateral flex due to the high carbon contents. This, however, can be remedied by post-growth processing, as discussed in the chapter “Materials and Functionalities”.

### 3.3. Applications

**Figure 4:**
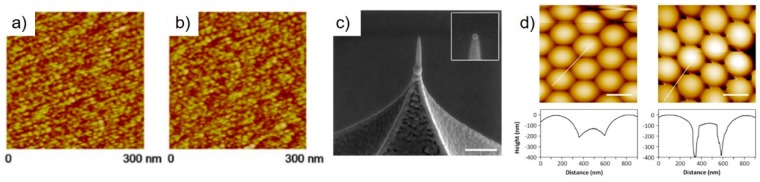
Atomic force microscopy (AFM) imaging with FEBID nano-pillars. (**a**) and (**b**) show tapping mode height images of SiN after 0.5 h and 7 h continuous operation, respectively, where the maintained image quality reflects the tip robustness. The Z-range is 3 nm in both high-resolution images. (**c**) shows a commercially available AFM tip, modified by a Pt–C nanopillar, revealing an end radius of ∼4 nm, as shown by a circle in the inset (scale bar is 200 nm). (**d**) shows a direct comparison of close-packed polystyrene spheres obtained with the aforementioned standard tip and the FEBID tip at the left and right, respectively (scale bar is 400 nm). Selected line scans along the white lines are shown below and clearly demonstrate the advantage of deeper profiling abilities via FEBID tips. (**a**) and (**b**) were adapted and reprinted from Chen et al., Nanotechnology 2006 [64], (**c**) and (**d**) were adapted and reprinted from Brown et al., Ultramicroscopy 2013 [28].

**Figure 5:**
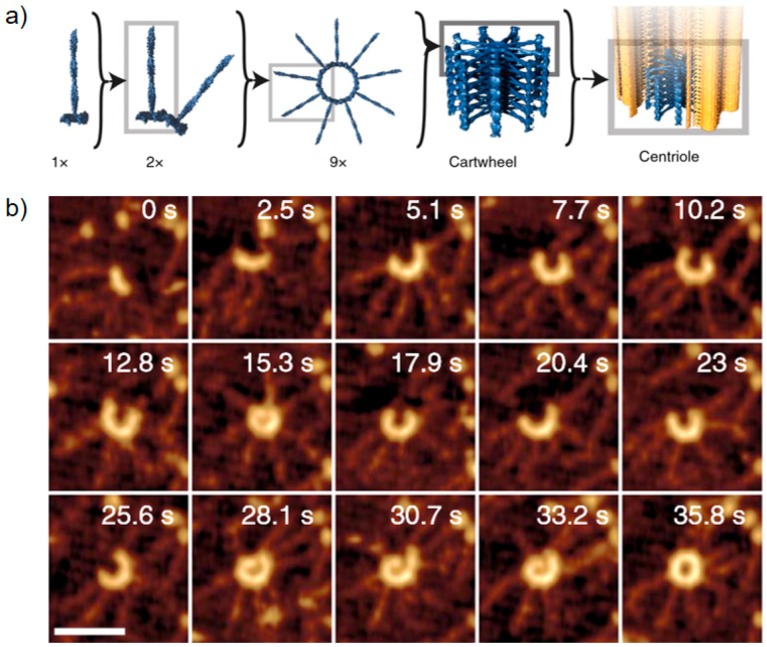
High-speed AFM imaging of biological systems using FEBID modified Si tips. (**a**) Formation process of cartwheels and centrioles by the assembly of CrSAS-6 homodimers, which arrange due to their coiled-coil/globular head domains. (**b**) High-speed, high-resolution imaging using FEBID tips, which make it possible to follow the formation of a cartwheel. Scale bar is 50 nm; Z range, 6.3 nm. Adapted and reprinted from Nievergelt et al., Nat. Nanotechnol. 2018 [65].

The performance of simple pillar-based tip modifications of SPM probes has been demonstrated by different groups. The first reports go back to 1993, by Schiffman et al., who demonstrated the superior characteristics of high aspect ratio FEBID carbon tips compared to conventional, pyramid-like AFM tips [61]. More recently, Chen et al. [64] showed the improved lateral resolution capabilities of such tips using mica, copper, and SiN samples in dry and, in particular, in liquid conditions, which maintain their quality even after 7 h continuous measurements (see Figure 4a,b). Using a similar FEBID tip modification approach, Brown et al. could clearly demonstrate the advantage of the high aspect ratio characteristics, as shown in Figure 4c [28]. In more detail, they used closely packed polystyrene spheres as test samples and could show that FEBID tips went about 200 nm deeper than commercial tips, which also improved the lateral diameter analyses for such challenging sample surfaces (see Figure 4d). Another very impressive application was shown by Nievergelt et al., who used FEBID-modified Si tips for high-speed measurements of biological systems [65]. Figure 5a shows the assembly process of coiled-coil/globular head CrSAS-6 homodimers in liquids. The system ends up in centriolar cartwheel arrangements, which act as scaffolds for the formation of a new centriole via the recruitment of additional peripheral components (shaded yellow). Figure 5b shows a time-resolved series of high-resolution measurements, which directly reveals the formation process of the first cartwheel. In that study, the application of FEBID tips was essential from an adhesion point of view, while high-resolution capabilities were indispensably needed and successfully demonstrated (scale bars are 50 nm). Further applications, which strongly benefit from the resolution capabilities of FEBID tips, are discussed later in the functionality context.

### 3.4. Challenges

The actual challenges in terms of feature size are mainly related to the cross-sectional shapes of individually inclined 3D-FEBID nanowires, especially when elements are not parallel to the incoming electron beam. As can be seen in Figure 1a, freestanding wires can reveal a blade-like character with the longer dimension of the vertical cross-section being oriented along the electron beam axis. Such non-circular shapes entail spatially inhomogeneous mechanical stiffness, which can become detrimental during AFM measurements, where the structure twisting or inhomogeneous bending convolutes the final image [66]. Perfectly circular shapes only arise for vertical pillars deposited via stationary exposure, while conversely, inclined segments reveal a non-linear variation of its thickness-to-width aspect ratio. These non-symmetrical cross-sections are highly correlated with the electron–solid interaction volume and therefore depend on the primary beam energy, the density of the deposited material and on the inclination angle. A very good compromise, however, is the application of low primary energies (around 5 keV and less), leading to thickness:width aspect ratios around 1.2:1 for a wide inclination angle range. The physics governing the shape of the inclined segment cross-section, and the scaling behavior, are currently under investigation and will be disseminated in a future publication. The long-term goal based on current research activities is a patterning-based compensation that enables close to circular cross-sectional shapes, independent of process parameters and inclination angles [67]. Subsequently, this capability will be extended to tune the cross-sectional shapes along single branches, which would open up entirely new possibilities for application-specific tailoring (e.g., mechanical, magnetic, or optical anisotropy in 3D space).

## 4. Materials and Functionalities

Apart from feature size aspects, the functionality becomes relevant when focusing on special SPM operation modes, such as electrostatic force microscopy (EFM), Kelvin force microscopy (KFM), magnetic force microscopy (MFM), or scanning thermal microscopy (SThM), to name a few. Consequently, the availability of suitable precursor materials is of essential importance. Figure 1b shows the periodic table and indicates elements that have successfully been used for FEBID (yellow and green) and 3D-FEBID (green). While a more detailed overview of metallic and magnetic material properties of FEBID materials is given by Huth et al. [23] and de Teresa et al. [24], here, we limit our discussion to 3D-FEBID structures.

With a few exceptions [8,24,68,69,70,71,72,73,74], FEBID materials notoriously suffer from high levels of contaminants (usually carbon) due to incomplete precursor molecule dissociation. The contamination accumulates as partially dissociated molecular fragments become incorporated in the deposit. The precursors are often organometallic in nature, which explains the high carbon levels observed [23,75]. The microstructure of such deposits reveals a nanogranular character, where metallic nano-grains (~2–5 nm) are embedded in a (hydro-) carbon matrix, as evident by dark- and bright-appearing regions in the TEM micrograph in Figure 2. Contaminated microstructures reduce or even entirely mask the intended functionality. Detailed studies concerning composition tunability using both primary beam parameters and patterning strategies revealed a limited ability to control contamination levels [76,77]. This issue was addressed in recent years by the successful introduction of several protocols to tune both chemical purity and functional properties [23,75]. For example, the functional properties can be tuned via post-growth electron beam exposure, denoted as e-beam curing (EBC) [78]. EBC initiates two processes: (1) the dissociation of incompletely dissociated precursor molecules, which release target atoms, leading to slight grain-growth [22,23,78,79,80]; and (2) the modification and cross-linking of the carbon matrix (sp^3^ to sp^2^) [21,29,80,81]. The former effect allows precise tuning of the electrical conductivity [78] and even enables a controlled insulator–metal transition, as demonstrated for Pt und Au [18,22,23,78]. The underlying process is the modulation of the Coulomb barrier due to reduced grain-to-grain distances, which enables chemical [39,82] or thermal sensing, as discussed later. The EBC cross-linking effect has mechanical implications, as the Young modulus can precisely be tuned, as demonstrated for Pt based materials [21]. Please note, EBC has a minor effect on the chemistry but a major effect on the (electric) functionality, which is extremely useful, as discussed later. In other cases, material purity is of highest importance. In recent years, different approaches have been demonstrated, including gas-/laser-/temperature-assisted approaches during [72,83,84,85,86,87,88] or after deposition [44,89,90,91]. Although different in execution, the common element for most of these approaches is the tendency for morphological disruption during carbon removal, which becomes even more challenging for freestanding 3D nano-architectures [44,84]. This aspect is discussed in the “Challenges” section below. In the following, we review different SPM applications, which take advantage of both its morphology and its material properties.

### 4.1. Applications

#### 4.1.1. General AFM

Carbon-containing 3D-FEBID structures exhibit high elasticity during force load [29], which originates from the high carbon content of up to 90 at.% [75], equivalent to more than 70 vol.%. During AFM operation, the tip experiences forces in vertical and lateral directions, which immediately implies mechanical properties of single pillars, which can be considered as fundamental building blocks for more complex 3D-FEBID architectures. This topic is reviewed in great detail by Utke et al. in another article of this special issue, which gives a comprehensive insight into mechanical properties, and their dependency on fabrication and on post-growth treatment approaches [92]. In brief, FEBID pillars are much stiffer along the main axis compared to situations where lateral forces become relevant [66]. As the latter depends on the pillar lengths as well, single-pillar-like FEBID structures should be as long as needed, but as short as possible.

EBC has been demonstrated to change the Young modulus from about 14 GPa to 80 GPa (even higher values are possible [92]), without affecting the overall morphology, including the apex radii [21]. The great advantage of EBC is the minimally disruptive influence, even on fragile 3D nanostructures, which makes this approach almost straightforward. Although the material gets stiffer by EBC, the risk of breaking at the interface during SPM operation remains and gets even higher, as arising forces get more concentrated to those regions [66]. The solution for this problem can be more complex 3D designs, where one conceptual example is shown in Figure 3b. In a recent study, the relationship between 3D design and the resultant stiffness in vertical and lateral direction was explored [66]. It was found that the tri-pod and tetra-pod arrangements strongly increase the stiffness—up to 60 N/m and 5 N/m in the vertical and lateral direction, respectively—which comes into the relevant range when aiming for SPM applications. The tetra-pod arrangement provided the best overall mechanical properties with radially symmetric stiffnesses. EBC treatment further increased the stiffness up to 200 N/m in the vertical direction, which is sufficient for stable AFM operation. In the same study, the advantageous implications of EBC treatments on the wear resistance of the tip apex could be shown, which finally allowed high-speed (~80 µm/s) and long-term AFM measurements (up to four hours were demonstrated without wear effects) [66]. Importantly, the study conclusively demonstrated the suitability of FEBID-based 3D nanostructures for AFM operation with high-resolution capabilities.

From a commercial point of view, FEBID-based tip modification has already found its way into commercial products by the company NanoTools (Munich, Germany), who provide a broad variety of shapes, dimensions, and fabrication angles, based on FEBID’s 3D capabilities [93].

#### 4.1.2. Electric

**Figure 6:**
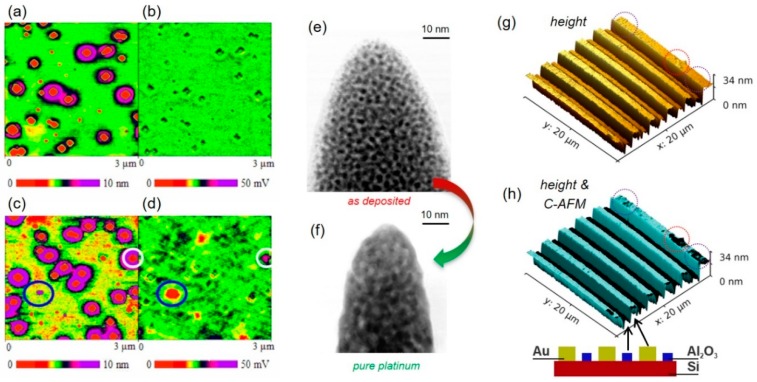
FEBID-based electric AFM modes. (**a**–**d**) show height (left) and Kelvin force microscopy (KFM) (right) images of SiGe quantum ring, performed with commercial, Pt–Ir coated AFM tips (upper row) and Pt–C nano-pillar modified tips (lower row). While the resolution improvement is evident by the more circular feature shapes, the KFM images reveal many more details, as representatively indicated by the blue and white rings. Adapted and reprinted from Chen et al., IEEE 2012 [94]. (**e**,**f**) show TEM micrographs of the tip region of a Pt-based FEBID nano-pillar after deposition (**e**) and after full purification using e-beam assisted carbon removal in H_2_O atmospheres at room temperature [44,90]. As evident, the pillar gets smaller in width, which entails a slight reduction of the apex radius in the sub-10 nm regime. The larger Pt crystals and the dense packing are evident, while the carbon-free character was confirmed by scanning transmission electron microscopy-based electron energy loss spectroscopy (STEM-EELS) measurements. Such highly conductive tips are then used for C-AFM measurements, as representatively shown in (**g**) and (**h**). The scheme below shows the layer setup, consisting of Au paths separated by Al_2_O_3_ lines on Si. The advantage of the high aspect ratio pillar is an accurate edge profiling in the height image (**g**), while the current signal (skin overlay in (**h**)) allows for the identification of non-conductive regions of thick (red circles) and nanometer thin (purple rings) impurity layers. Image copyright GETec Microscopy 2019 [95].

Next, we focus on electrically-based SPM modes such as C-AFM, EFM, and KFM. The earliest reports on the application of FEBID nano-pillars as electrically conductive tips go back to the early 1990s, where Hübner et al. demonstrated that even carbon tips can be used for STM [60]. Shortly after, Schössler et al. delivered one of the first reports on the application of a metal-organic precursor for STM tip modification. In more detail, an Au-based precursor was used for the fabrication of field emitters with sub-10 nm apex radii and an angular emission density of 0.2 mA/sr, which were successfully applied as STM tips and as STM-based nano-lithography tools [16]. The latter application was also demonstrated by Wendel et al., who used FEBID-based, ultrahard, amorphous carbon tips for nano-lithography. By a combination of hard tapping and triangular voltage pulses, they could fabricate 25 nm lines (FWHM) in photoresist, which finally allowed the fabrication of 30 nm metal structures [17]. More recently, Chen et al. modified commercially available, Pt–Ir coated conductive AFM tips by 20 nm wide and 250 nm long, Pt-based FEBID pillars [94]. The tips were then used for EFM and KFM measurements of Si–Ge quantum rings, where they could reveal the existence of a central Si–Ge-like area within the quantum rings themselves, as shown in Figure 6a–d. The improved lateral resolution was due to the small pillar diameters and, in particular, due to the small apex radii for FEBID pillars (sub 10-nm; see Figure 2a), which is about 2–3 times smaller than coated Si tips (nominally specified with 20–25 nm). That situation is shown in more detail in Figure 3c, which is a tilted SEM image of a coated tip (red arrow), further modified by a Pt FEBID-nano-pillar (green arrow). While the much smaller apex radius for the latter is immediately evident, Figure 3d shows a direct comparison of AFM height images on an Au nano-particle sample in contact mode. The improved lateral resolution achievable with FEBID tips (upper) compared to Pt–Ir coated tips (lower) relies on the fact that fully purified FEBID tips do not have/need a conductive coating, which makes them inherently sharper. While Chen et al. successfully demonstrated the EFM/KFM suitability for FEBID tips even in their as-deposited state, C-AFM requires fully purified (metallic) tips for reliable measurements. As mentioned above, different protocols have been introduced, which allow the chemical transfer into carbon-free FEBID materials. A particularly gentle approach is the electron beam-assisted purification in an H_2_O atmosphere (20–120 Pa) at room temperature, which has successfully been applied to Pt- and Au-based FEBID materials [44,83,90]. The chemical purification approach is highly efficient and creates defect-free, concerning nano pores or cracks, structures after purification. Figure 6 shows a direct, in-scale comparison of a Pt–C nano-pillar before (e) and after full purification (f), which shows: (1) the reduced apex radius, (2) increased Pt grain sizes, (3) dense grain packing, and (4) the maintained shape fidelity. Complementary scanning transmission electron microscopy-based electron energy loss spectroscopy (STEM-EELS) measurements confirmed the carbon removal under ideal purification conditions [44,90].

Such tips can then be used as C-AFM tips, which is exemplarily shown in Figure 6g,h, where the surface topography is revealed for a multi-layer structure, as schematically shown at the bottom. While the height image (g) takes advantage of both the sharp apex and the steep side walls to reach the bottom at fine trenches, (h) shows a 3D height plot with the current signal as overlay. As evident, the correlated measurements are almost digital, revealing conductive (turquoise) and insulating (black) regions and identifying nanometer thick surface impurities (purple circles). Apart from resolution aspects, there is another essential advantage of fully purified FEBID tips compared to coated AFM tips: because they are entirely made of pure material (e.g., Pt), the delamination problem of coated AFM tips is entirely absent, also making such probes more durable. Such FEBID-based conductive tips will then fulfill the requirements for application in C-AFM, KFM, and EFM, as already shown conceptually, but can also be used for piezoelectric force microscopy (PFM) and scanning spreading resistance microscopy (SSRM). Notably, Noh et al. [96] and Roberts et al. [97] have previously used FEBID and focused electron beam-induced etching (FEBIE) for the fabrication of advanced insulated PFM and SPM probes for use in liquid environments. In short summary, the particular advantages of FEBID tips for electrically-based AFM modes are the sharp apex, high aspect ratio shapes if needed and the proven wear resistances. Beyond that, FEBID allows for special designs, which enable morphological adaption according to the individual situation, as discussed in the “Design Flexibility” section below.

#### 4.1.3. Magnetic

Now, we turn to magnetic tips, where the material quality is critically important to provide the required sensitivity during MFM measurements. Iron and cobalt are the most promising candidates for precursor materials, based on Figure 1b. As-deposited Fe and/or Co FEBID purity must be high for the ferromagnetic property to emerge, as discussed in a review by de Teresa et al. [24]. To purify magnetic materials, H_2_O or O_2_ assisted approaches are not suitable, because surface and internal oxidation occurs [24]. Hence, different research groups have explored the process window during initial FEBID to minimize unwanted impurities. The first studies in that direction stemmed from Utke et al. and Lau et al., who fabricated nano-pillars from Co_2_(CO)_8_ precursor for MFM tests [32,98,99]. The Co nano-tips were composed of 34 at.% Co, 51 at.% C, and 14 at.% O and allowed a lateral resolution in the same range as for commercially available, coated magnetic tips (~40 nm), but provided the high aspect ratio advantages, as discussed before. In the following decade, strong progress was made in the fabrication, optimization, and application of magnetic materials in general, as comprehensively reviewed by de Teresa et al. [24]. Several studies explored the variation in chemical composition as a function of primary electron beam conditions [100,101], patterning parameters [102], gas flux implications [103], and substrate temperatures [101,104]. Co contents up to 95 at.% were found at higher beam currents, primary energies, and elevated temperatures, where the remaining C/O fraction stems from the unavoidable surface oxidation, once the sample is exposed to ambient conditions after processing.

**Figure 7:**
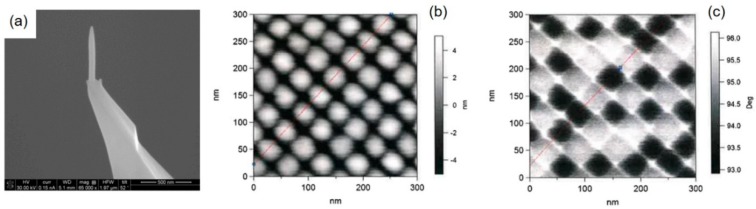
Sub-10 nm resolution magnetic force microscopy (MFM) imaging via FEBID super-tips. (**a**) shows a Co-based, FEBID super-tip with a metal content of ~60 at.% and end radii below 10 nm. Such tips are then used for imaging bit patterned media, as shown by the height and lift-mode phase images in (**b**) and (**c**), respectively. As evident, the MFM image (**c**) clearly reveals the written bits with lateral resolution in the sub-10 nm regime. Adapted and reprinted from Belova et al., Rev. Sci. Instrum. 2012 [111].

For iron-based materials, the most relevant precursors are Fe(CO)_5_ and Fe_2_(CO)_9_, first studied by Takeguchi et al. [105] and Furuya et al. [106]. In similar studies as those reported above for Co, primary beam and patterning parameters, H_2_O co-exposure, substrate temperatures, and post-growth annealing steps were studied for Fe-containing deposits (see reference [24] for a detailed overview). Particularly interesting was the study by Takeguchi et al [105], in which FEBID’s 3D capabilities were used to study the relationships between morphology, thermal post-treatments, and the resulting magnetic properties [105]. That study was one of the first which applied FEBID’s powerful 3D capabilities, by means of rods, dots, rings, triangles, and even more complex structures, for fundamental research. A particularly important study was done by Rodriguez et al. [107], who investigated the coercive field as a function of width and thickness in Fe nanowires. The findings clearly revealed that narrow tips below 50 nm in width should be highly beneficial for MFM applications, while the thickness is not as critical. A similar result was found for Co-based nanowires, as reported by Venturi et al. [108] and Wolf et al. [109], who studied the magnetic induction as well as magnetic stray fields as a function of the tip distance for full optimization of MFM tip morphologies. Design rules were proposed as a result of these studies, which showed that the tip lengths can be adapted according to the requirements (pinholes, sidewalls, grooves), while the tip diameter should be as small as possible. Gavagnin et al. [42] confirmed these findings experimentally and also demonstrated the importance of the relative tilt angle between tip and sample, which has to be perpendicular for the highest lateral resolution and magnetic sensitivity. From an application point of view for Co-based FEBID structures, Stiller et al. reported the modification of Akiyama tips [110], while Belova et al. demonstrated the optimized fabrication of Co-based FEBID super-tips as shown in Figure 7a. The metal content of those tips was around 60 at.%, and allowed lateral resolutions down 10 nm, which can be considered as the current benchmark for FEBID-based MFM tips [111].

While FEBID-based Co structures can reveal metal contents up to 95 at.% right after deposition, Fe-based nanowires typically range around 50 at.%, which is too low to exploit their full potential. Jose-Maria de Teresa et al. demonstrated that such Fe-based nanowires can be transformed into nominally pure, highly crystalline, and morphologically stable nano-pillars via thermal post-growth annealing at temperatures of at least 400 °C [105,112]. The latter, however, might be challenging for cantilever platforms, which contain integrated components, such as self-sensing or resistive elements.

**Figure 8:**
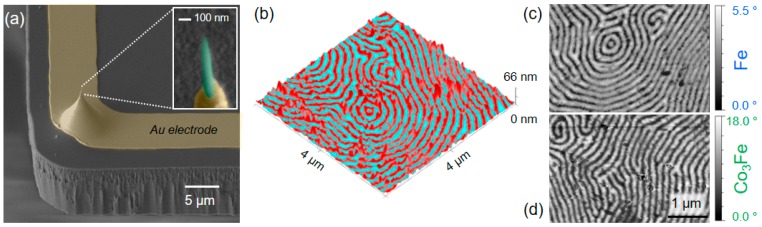
FEBID-based, high-performance MFM tips. (**a**) shows a tilted SEM side view of a pre-structured, self-sensing cantilever, modified by a magnetic Co_3_Fe tip via 3D-FEBID at 20 keV/13 pA. Such tips reveal typical diameters and apex radii well below 100 nm and 10 nm, respectively. (**b**) shows a 3D height image with the MFM signal as overlay, taken from a Co/Pt multilayer structure with perpendicular magnetic anisotropy. (**c**) and (**d**) show MFM phase images from the same sample, however, acquired via Fe and Co_3_Fe FEBID tips, with similar morphologies. A closer look reveals that MFM tips from Co_3_Fe reveal much stronger phase contrasts by a factor of more than three, while lateral resolution is also improved to the sub-20 nm regime. Images are courtesy of GETec Microscopy [95] (**a**–**c**) and Michael Huth (**d**). Copyright 2019.

A heteronuclear Fe–Co precursor (HCo_3_Fe(CO)_12_) was synthesized and demonstrated to be fully 3D-FEBID compatible, which serves as an alternative to the homonuclear paradigm [113,114,115]. The resulting deposits revealed a Co_3_Fe composition, which is nominally carbon-/oxygen-free when disregarding the thin, unavoidable surface oxidation layer. This precursor material is an ideal candidate for MFM tip modification owing to the low vaporization temperature and non-reactive character with the surface. Figure 8a shows an SEM side view of a self-sensing cantilever with a pre-existing tip, modified by a Co_3_Fe nano-pillar, shown in more detail at the right. Such tips reveal typical FEBID diameters and apex radii well below 100 nm and 10 nm, respectively, which can be used directly after fabrication without any further post-growth treatment. For benchmark purposes, a Pt/Co multilayer system was studied, as representatively shown in Figure 8b by a 3D height scan with an MFM phase overlay. The perpendicular magnetic anisotropy leads to a clear magnetic phase contrast, not recognizable in the morphology. Figure 8c shows the phase contrast in more detail, using an Fe-based FEBID nano-pillar, fabricated at optimized growth conditions. Individual features are well resolved, while typical phase shifts range around 5° for optimized lift heights and amplitudes. In contrast, Figure 8d shows the same sample imaged by a Co_3_Fe super-tip with very similar overall dimensions. As clearly evident, phase shifts are more than three-times higher at optimized lift heights (see scales), which reduces the noise and increases the lateral resolution, which was found to be clearly below 20 nm. Together with the fact that growth times are similar, these first attempts show the high performance of this heteronuclear precursor. Rounded up by the uncomplicated handling, this material has enormous potential to revolutionize FEBID-based fabrication of magnetic tips for high-performance MFM operation.

**Figure 9:**
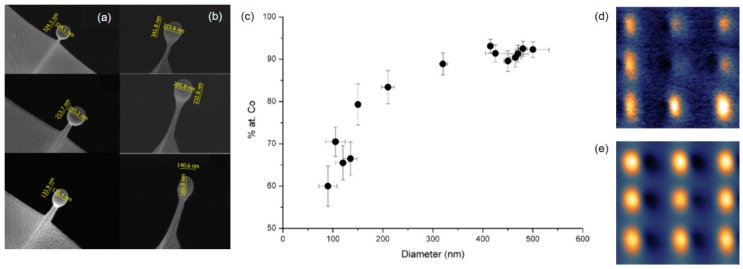
FEBID-based ferromagnetic resonance force microscopy (FMRFM). (**a**) and (**b**) show differently sized Co nano-spheres (top down), fabricated on top of a high-resolution AFM cantilever in the top (**a**) and side views (**b**), revealing a close to spherical morphology. (**c**) shows the cobalt content of the beads as a function of the diameter, which has to be correlated with its magnetic properties. Additional measurements revealed a minimum diameter of 150 nm to exploit the full performance. (**d**) and (**e**) show FMRFM and MFM measurements, respectively, using a Co-AFM cantilever modified via FEBID. These measurements use an in-plane magnetic field, which aligns the sample magnetization. A microwave-frequency current is then introduced perpendicular to the magnetic field, but also in-plane. The latter decreases the quasi-static component of the magnetization, which impacts the magneto-static force between the sample and tip. Once the magnetic resonance is found, FMRFM reveals defects induced by chemistry or morphology, as provoked in this example by a different shape of the central element in the 3 × 3 array. This explains why that part is dark in FMRFM mode (**d**). In contrast, classical MFM measurements are unable to detect such defects, as shown in (**e**). Although powerful in its analytical capabilities, the lateral resolution is currently limited to about 90 nm, as that depends on the bead diameter. (**a**–**c**) were adapted and reprinted from Sangiao et al., Beilstein J. Nanotechnol. 2017 [117]. (**d**) and (**e**) were adapted and reprinted from Chia et al., Appl. Phys. Lett. 2012 [118].

Apart from these classical applications, more advanced AFM operation modes have been demonstrated in combination with FEBID tips. A powerful expansion to classical MFM measurements via phase-shift detection is ferromagnetic resonance force microscopy (FMRFM). This operation mode is based on the detection of the magnetic resonance with a magnetic sample due to the magnetic force exerted on a cantilever equipped with a small magnetic tip [116]. Sangiao et al. fabricated small Co nano-spheres via FEBID on top of a soft AFM cantilever in a very controlled manner, ranging from 100 nm to 500 nm, as shown in Figure 9a,b [117]. The spherical shape is advantageous for FMRFM operation, as it minimizes the magnetic hysteresis. The Co content was revealed to increase with the diameter (Figure 9c), while a minimum diameter of 150 nm was found as the lower limit for FEBID–Co spheres for appropriate magnetic sensitivity [24], almost eliminating unwanted remanence effects. Although the lateral resolution during FMRFM operation correlates with the size of the nanosphere, impressive results have been shown in recent years [118,119,120,121,122,123]. Figure 9d,e shows a representative result, in which FMRFM was used for defect detection (in this case, morphologically induced), while classical MFM operation is unable to extract that information (compare (d) with (e)).

#### 4.1.4. Thermal

**Figure 10:**
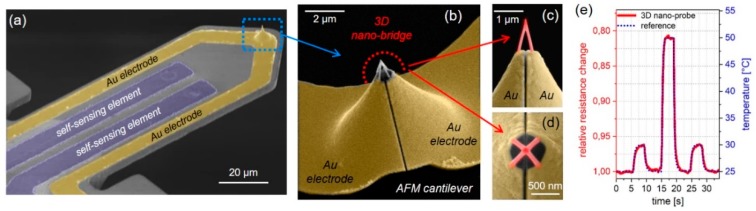
FEBID-based 3D nano-probes for localized thermal sensing. (**a**) shows the self-sensing cantilever platform (copyright GETec Microscopy, Austria [95]), where sensing elements and pre-structured Au electrodes are indicated blue and yellow, respectively. (**b**) shows an SEM image of the tip region, in which the truncated, pre-existing tip, the split electrodes, and the 3D nano-bridge are evident. The latter is shown in higher magnification in a side (**c**) and top view (**d**), where the 3D nano-bridge is shown in red. Once in contact with the sample surface, the small active volumes of the 3D structure allow a fast response, as shown in a dynamic response test in (**e**) by the red curve in comparison to the reference temperature (blue). A sensing rate better than 30 ms/K with a noise level below ±0.5 K could be demonstrated, which exhibits the advantages of 3D-FEBID nano-structures for advanced AFM concepts, applicable on even pre-finished micro-cantilever. Adapted and reprinted from Sattelkow et al., Appl. Mater. Interfaces 2019 [66].

As mentioned before, EBC has a minor influence on deposit morphology but a strong impact on the electrical function: the nano-granular composition remains, while the electrical tunneling probability between adjacent nano-grains increases [23,78]. The latter effect also depends on temperature, leading to decreasing resistances at higher temperatures due to thermally assisted tunneling. By that, the macroscopic current through such bridges provides information of the material’s temperatures with a negative temperature coefficient (NTC) of electric resistance. This thermistor-based SThM concept was first shown by Edinger et al. in 2001 [124,125], but not followed in more detail, which might be explained by the limited knowledge about material tuning and controlled 3D nano-printing capabilities at that time. Sattelkow et al. recently revisited the 3D thermistor concept, optimized mechanical/electrical aspects, and demonstrated thermal probing [66]. As summarized in Figure 10, the authors used a self-sensing AFM cantilever platform (a), which was pre-structured with two electrodes, covering the pre-existing tip as well (b). FEBID tetra-pods were used as 3D nano-bridges between those two electrodes, as shown by an overview (b) and by close-ups in (c) and (d). Once in contact with the sample, the tetra-pods heat up and change their electric conductivity due to the NTC behavior. Because of the small active volumes in the FEBID-created contact region, the heat response is very fast and allows for dynamic sensing of at least 30 ms/K, as shown in Figure 10e. This functional nano-probe not only demonstrates AFM compatibility but also the usefulness of a nano-granular material, a microstructure which is often regarded as ineffective concerning real applications. This hallmark demonstration of functional transduction was complemented by an apex-radius below 10 nm and fast fabrication times around 10 minutes, including EBC.

#### 4.1.5. Optical

A first example of FEBID-based spectroscopy was reported by Castagne et al. in 1999, who modified Si–AFM probes by carbon supertips with variable lengths (1.3–3.3 µm), widths (130–300 nm), and apex radii down to 15 nm [126]. Such probes were then used in a combined AFM/photon scanning tunneling microscope to study their near field optical behavior. The study revealed that even carbon tips are convenient for near field optical conversion of evanescent waves in the near infra-red range. The authors also stated that the lateral resolution during imaging may be limited as a consequence of the optical properties of carbon tips and gave theory-based recommendations for further optimization towards high-performance optical near field nanoprobes for application in scanning near field optical microscopy (SNOM) or TERS microscopy. Shortly after, Sqalli et al. focused on the beneficial implications of small FEBID-based Au nano-dots/-ellipses on light scattering capabilities during SNOM [127]. In more detail, light fiber-based probes were modified by differently shaped FEBID nano-structures and studied by a combination of theory and experiments. The authors could demonstrate that spherical structures with ~60 nm diameter increase both the signal and the contrast in near field transmission measurements. The study also showed that elliptical shapes allow tuning of the resonant wavelength as a consequence of the relative orientation between elliptic nano-structures and the incoming polarized light, which demonstrates FEBID’s usefulness due to its design flexibility.

**Figure 11:**
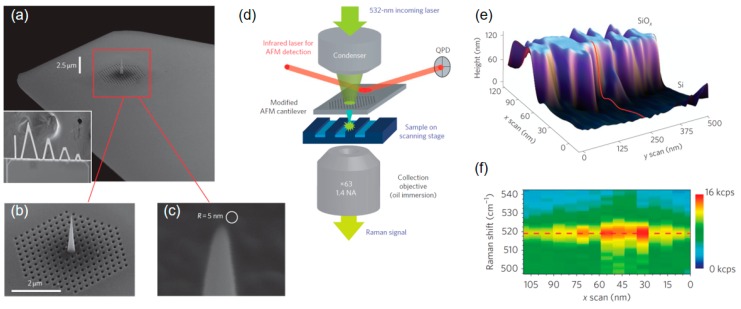
FEBID/ focused ion beam (FIB) assisted, photonic/plasmonic tip-enhanced Raman scattering (TERS) nano-probe. (**a**) Overview of a 100 nm thick Si_3_N_4_ AFM cantilever, equipped with the photonic and plasmonic element, based on FIB and FEBID, respectively. (**b**) shows a close up in which the FIB holes (160 nm diameter and 250 nm distance) are evident. The central element uses a Pt-based 3D-FEBID pillar as scaffold, further coated with pure Ag and milled down via FIB to apex radii of 5 nm and below (**c**). Such TERS nano-probes are then used in AFM-based configuration, as shown in (**d**), where the laser couples in from the backside via the photonic element, launching surface plasmons. After propagation along the cone structures, they induce a localized plasmon resonance at the tip apex in a nanometer-sized volume. (**e**) shows an AFM height image in 3D representation of a sub-micrometer Si nanocrystal/SiO_x_ trench. The Raman intensity along the red line is shown in (**f**) in a spectrally resolved diagram, where the intensity variation reflects the crystallinity degree. As evident, the applied step size of 7 nm allows for sharp intensity edges, which confirms a sub-10 nm resolution in TERS operation. Adapted and reprinted from De Angelis et al., Nat. Nanotechnol. 2010 [128].

An impressive study concerning TERS microscopy was done by De Angelis et al., who introduced a highly advanced TERS tip concept, based on focused ion beam (FIB) and FEBID [128]. Figure 11 shows the tapered waveguide, fabricated on a 100 nm thick Si_3_N_4_ AFM cantilever (a). First, FIB was used to fabricate the photonic crystal, consisting of a triangular region with hole and separation distances of 160 nm and 250 nm, respectively (b). For the central waveguide, a Pt-based, 3D-FEBID structure acted as scaffold, which was covered with 30 nm Ag and down-shaped via FIB (c) to apex radii between 2.5 and 5 nm (further process steps were applied, as detailed in reference [128]). Such probes were then used in AFM operation, where the incoming laser is coupled via the backside through the photonic element, which launches surface plasmon, further propagating along the Ag tip to the apex, where the localized plasmon resonance occurs (d). Detection of the Raman signal was done in transmission. Figure 11e,f show an AFM 3D height image of a sub-micrometer Si nanocrystal/SiO_x_ trench and the corresponding Raman intensity along the red lines, respectively. The varying intensity reflects the crystallinity of the material, which correlates well with the wall region (e), which proves sub-10 nm resolution. While FEBID structures acted as scaffolds in this case, recent progress concerning purity and shape stability hold the potential to fabricate the central tip solely via 3D-FEBID [44], which would considerably simplify the entire fabrication process.

**Figure 12:**
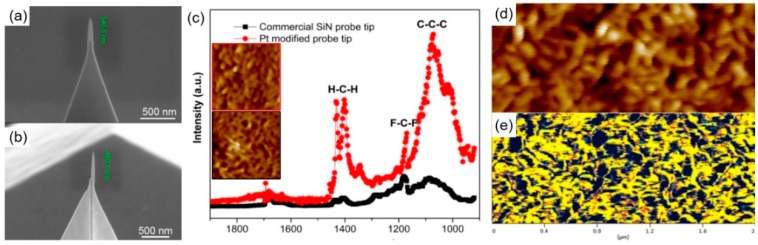
FEBID-based AFM-IR. (**a**) and (**b**) show two examples of Pt- and W-modified cantilevers, respectively, which were further treated via e-beam curing (EBC) to increase the electric conductivity for the required electric field enhancement during IR operation. (**c**) shows a comparison of localized nano-IR spectra on thin poly-vinylidine-fluoride (PVDF) films, performed via commercial SiN (black) and FEBID modified tips (red), where the enhancement in chemical sensitivity is clearly evident. The insets show 1.5 µm wide height scans (same frame color as the spectra), which reveals the improved lateral resolution. (**d**,**e**) show the 2 µm wide correlated height and chemical mapping image of PVDF, respectively, acquired at a constant wavenumber of 1078 cm^−1^, which indicates the asymmetric C–C stretching mode in the β-phase and allows morphology to chemistry studies. Adapted and reprinted from Qian et al., Nanotechnology 2018 [129].

Another very interesting application was demonstrated by Qian et al., who used FEBID-modified cantilever tips for AFM-based infrared spectroscopy (AFM-IR) on two polymer test systems [129]. Specifically, the authors modified SiN_x_ cantilevers with Pt- and W-based FEBID tips with lengths of 240–460 nm, diameters around 80 nm, and apex radii down to 12 nm, representatively shown in Figure 12a,b. Such tips were then subjected to EBC treatments, which slightly reduced the carbon content, but strongly improved the electrical properties, which are needed for the required electric field enhancement to increase chemical sensitivities. The study clearly showed the improvement of (1) lateral resolution due to FEBID’s feature size advantages (insets in Figure 12c), (2) durability, and (3) sensitivity concerning localized nano-IR spectra, as shown in Figure 12c. The authors also demonstrated chemical mapping on polymers, which allows the identification of different material phases, as shown by the correlation of simultaneously gathered scans in Figure 12d,e. For optical applications, the tip shape is the crucial element, as it dictates the localized electric field enhancement. Maintaining the tip performance, however, can be complicated, as SNOM, TERS, and AFM-IR are mostly performed in contact mode, which can lead to morphological wear effects. In this context, a study by Sanchez et al. should be mentioned, who studied the optical performance of Si_X_O_Y_ coatings using focused ion beam induced deposition (FIBID) and FEBID from the tetraethyl-orthosilicate (TEOS) precursor [130]. The authors coated apertureless tips via both approaches by sub-10 nm layers, where FEBID resulted in smooth coatings with chemistries close to SiO_2_. More importantly, high spatial resolution during fluorescence spectroscopy without significant quenching was demonstrated, which might be a successful coating route to increase the wear resistance of all FEBID tips for optical SPM modes.

In summary, this section reviewed the most important SPM modes, which benefit from FEBID SPM tip editing, which includes small feature sizes, chemical purity, and morphological tuning. As a whole, the above demonstrations highlight the fast and precise modifications possible using FEBID, which requires only an SEM equipped with a GIS or, alternatively, a dual-beam microscope. The direct-write character is of particular relevance, as it allows the modification of pre-existing tips or full fabrication on a pre-finished cantilever system with the possibility to change the design in a fast and flexible way.

### 4.2. Challenges

Many precursors that are suitable for conventional FEBID (see Figure 1b, orange) have not been tested yet concerning their suitability for 3D-FEBID. Apart from strong autocatalytic growth effects or very low precursor fluxes, there are no real reasons why the fabrication of freestanding structures should not be possible with these precursors. A particularly interesting material for plasmonic applications is silver, which has recently been demonstrated to be 3D compatible [73]. For this special precursor, the authors identified the GIS alignment and proper heating of both the GIS and the substrate, as decisive elements, which currently are in an optimization phase to achieve sub-100 nm, 3D nanostructures via FEBID. Admittedly, 3D-nanoprinting of insulating materials (e.g., SiO_X_) is more challenging due to the increased charging and heating of the 3D object, although complex geometries have been demonstrated [34]. Currently, significant effort is being put on the development of new FEBID precursors (EU-ITN project ELENA [131]) to expand the portfolio of available materials. In parallel, there are challenges in tuning the chemistry and by that the functionality of as-deposited 3D objects by post-processing steps. In particular, the volume loss during the post growth purification, which can be ^2^/_3_ of the original volume, often leads to distortions or even to collapse of the original 3D-geometry. Although the structural integrity can be maintained by gentle purification conditions [44], the situation often has to be adapted from geometry to geometry, which is time consuming and partly cumbersome for challenging 3D architectures. While the situation is currently approached by different strategies, the ideal case would be precursor materials, which provide contamination-free deposits right after growth, as demonstrated for Co_3_Fe [23,113,114,115]. An alternative strategy is to purify the material along with deposition, which was partly successful by simultaneous injection of water vapor [83] or laser pulse cycles [84]. The drawbacks, however, are a higher partial pressure, which has not yet been demonstrated to be compatible with 3D-FEBID, or the high local temperatures in the case of laser pulses, which entails higher demands on the used substrate. Aside from the purity, multi-material 3D-FEBID is a highly interesting but challenging task. The idea to integrate different material properties in 3D-space (e.g., separation of conductive/insulating or magnetic/non-magnetic parts [113]) might open up new applications beyond the area of SPM, as discussed in the next section.

## 5. Design Flexibility

In the previous sections, AFM tips were mostly modified by single pillars. For novel operation modes, the fabrication of more complex designs at the tip region are needed, as discussed before for FEBID-based SThM tips [66]. The integration of more complex tip shapes by traditional fabrication methods would require, if possible, several lithographical and etching steps, which is time-, and thus, cost-intensive. To reduce the efforts, 3D-FEBID is an ideal approach, as it not only allows fabrication at almost any region, but also provides a very high design flexibility in 3D space [34,44,49,132]. FEBID’s potential for specifically-shaped AFM tips and even more advanced applications was already recognized in the early years of 3D-FEBID [133]. A particularly impressive example was demonstrated by Ooi et al. in 2000, who used 3D-FEBID for the fabrication of needles, hooks, and cages for the manipulation and characterization of single DNA fibers [134]. In the following, we review more recent examples, which use FEBID’s 3D capabilities and complement the section with a perspective on possibilities and remaining challenges.

### 5.1. Applications

**Figure 13:**
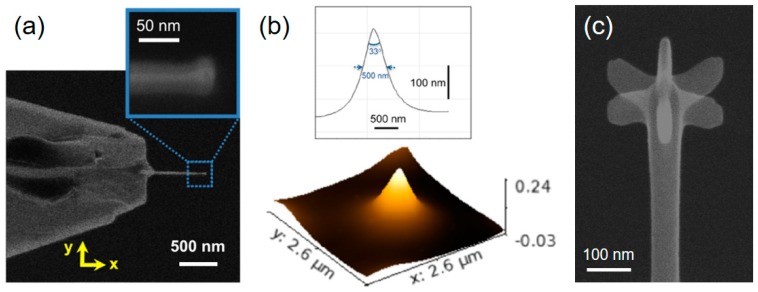
Non-conical apex design. (**a**) shows a truncated standard AFM tip, which was modified via Helium ion beam-induced Pt–C deposition, consisting of a 14 nm wide pillar, equipped with a hammerhead feature at the end (see upper inset). Please note, the pillar was laterally grown to reduce the width to a minimum, as shown for FEBID in Figure 3a. Such fine tips are then used for imaging shark fin structures, as shown by a 3D height scan in the lower part of (**b**). A corresponding cross-sectional profile is shown above, which reveals a measured opening angle of 33° over a height of more than 200 nm, which is very close to the real opening angle of 30°. To produce azimuthally equally distributed features at the apex, vertical growth modes are preferred, as representatively shown by the tilted SEM image of a 3D-FEBID structure in (**c**). This vertically grown tip consists of a 65 nm wide pillar, which is equipped with six radially symmetrical distributed blades with lateral expansions around 90 nm and widths of about 25 nm. At the top, a fine cone is protruding for localized detection of vertical sample interactions. (**a**) and (**b**) were adapted and reprinted from Nanda et al., J. Vac. Sci. Technol. B 2015 [137].

3D-FEBID makes it possible to design a wide variety of 3D architectures that can be used to address individual problems. As already mentioned, the weak connection of pillars to the tip/substrate is an issue, which can be minimized by the enlargement of the contact area by meshed or closed shapes, such as multi-pods (Figure 3b and Figure 10) or conical structures, respectively. While such approaches improve the overall mechanical integrity, the design of the apex region is of particular relevance, as it dictates the sidewall accuracy for very steep features during AFM imaging. An interesting approach to tackle this problem was demonstrated by Dai et al., who glued one and even two small AFM cantilevers on top of a larger AFM tip [135]. By that, they were able to map the surface characteristics of vertical walls. However, the fabrication route is not straightforward, and furthermore, specific adaptions are barely possible. For such situations, 3D-FEBID is ideal, as it provides both high design flexibility and a rapid prototyping character due to its direct-write character. The latter was applied by Matsui et al. in 1992, who first modified an STM tip by a vertical pillar, further modified by a laterally grown pillar. By that, they could demonstrate STM-based mapping of almost vertical side walls, including the side wall roughness [136]. Although not fabricated by electron beams but via focused He ion beams, Nanda et al. introduced a similar pillar-based tip modification, which was equipped with a hammerhead-like design at its end, as shown in Figure 13a [137]. Such hammerheads were then used for AFM characterization of deep but narrow trenches in Si and shark fin features. The latter is shown in Figure 13b by a 3D height image (bottom) and a related cross-section above. As evident, the measured side wall angles were around 73° compared to a reference angle of 75°, which means a deviation of less than 2° over a height of 250 nm. While He ion beam-based deposition is known to provide smaller feature sizes than FEBID [138,139], it should be mentioned that the extremely narrow pillar of 14 nm was achieved via horizontal growth, for a dimension that has been demonstrated via 3D-FEBID as well (see Figure 3a, revealing a pillar width of about 18 nm). While the current benchmark for such FEBID tips lies in the range of 5 nm with special protocols [8], the fabrication of sub-20 nm tips via horizontal growth is a routine application. In contrast, Figure 13c shows a vertically grown 3D-FEBID structure with six lateral fins, each less than 100 nm wide and high, with blade thicknesses around 25 nm. The central top tip is then responsible for the lateral resolution at the bottom, the fins allow profiling of deep vertical trenches. Such designs can be further optimized by decreasing the fin heights while increasing the fin width, which would then allow the profiling of undercuts, which is a major limitation of classical AFM probes.

Apart from classical SPM applications, 3D-FEBID based tips have also been used in different, but creative ways. A first example of mechanical-based lithography goes back to 1998, where Irmer et al. demonstrated the application of diamond-like FEBID carbon tips as nano-ploughs. By applying a high vertical force around 40 µN, they could plough 50 nm wide lines in a 300 nm thick Al film to realize highly transparent Josephson junctions [140].

**Figure 14:**
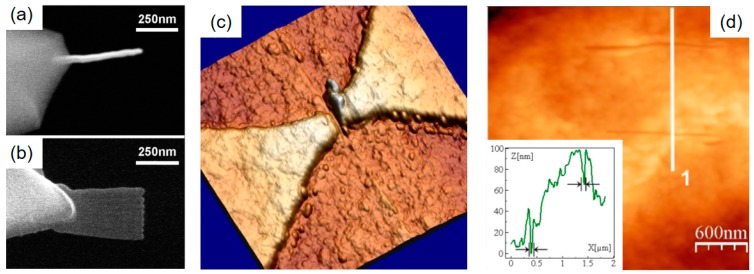
FEBID nano-scalpel. (**a**) and (**b**) are the top and side view SEM images of laterally grown 3D carbon structures, which evolve into blade like structures along the Z axis. Such blades have very sharp end regions with knife-like morphologies, which allow operation as nano-scalpel by modulating the exerted force. (**c**) shows the fabrication of a sub-25 nm wide gap across an Au electrode system by 3D height images, while (**d**) shows an example of a “nano-surgery” in smooth rat aortic muscle cells with constant cut widths of 50 nm, as shown by the cross-section inset at the bottom. Adapted and reprinted from Beard et al., Nanotechnology 2009 [141].

**Figure 15:**
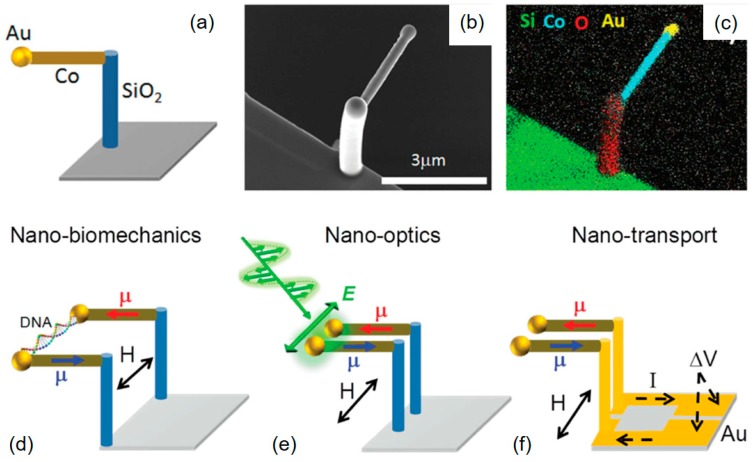
Application possibilities of Au/Co/SiO_X_ multi-material, nanoactuated magnetomechanical systems (NAMMS). (**a**) Basic design of a Co/SiO_X_ NAMMS device, equipped with a small Au sphere at the end of the moving Co cantilever. (**b**) and (**c**) show an SEM image and the correlated energy dispersive X-Ray analysis (EDXS) based elemental map, respectively, which demonstrate FEBID multi-material fabrication capabilities within a single 3D structure. (**d**–**f**) show potential application examples for correlated analyses concerning magnetic, mechanical, optical, and electrical properties at the nano-scale. Adapted and reprinted from Vavassori et al., Small 2016 [144].

Using a similar approach, the work group around Gordeev has used 3D-FEBID for the fabrication of nano-scalpels on top of standard AFM tips, as representatively shown in Figure 14a,b. In a series of publications, they not only demonstrated the controlled scalpel fabrication but also the tuning of their mechanical properties [29,141,142]. The experiments included lithographic applications for the formation of sub-25 nm gaps in Au electrodes and the controlled and precise incision in fixed rat aortic smooth muscle cells with constant cut widths of 50 nm, both shown in Figure 14c,d, respectively. While that study used the AFM as force transducing element, 3D-FEBID structures can also be used for nano-electro-mechanical-systems (NEMS) concepts, as demonstrated by Bøggild et al., who applied 3D-FEBID for the fabrication of sub-25 nm gap nano-tweezers [143]. In a more recent work, Vavassori et al. reported about multi-material 3D-FEBID for the fabrication of nanoactuated magnetomechanical systems (NAMMS), which were used initially as remote-controlled nano-tweezers. The high precision, demonstrated by the authors, actually forms the basis for more complex, truly unique applications in the area of nano-biomechanics, nano-optics, and nano-transport, as conceptually summarized in Figure 15 [144]. In principle, such concepts pave the way for further nano-electro-mechanical-systems (NEMS) for integration on SPM cantilevers, as such systems inherently provide extremely high navigation precision, especially when integrated in SEMs or FIBs for direct guiding. This would considerably expand the functionalities of SPM cantilevers with spectroscopic information, such as chemical sensing, demonstrated via 3D-FEBID resonators by Arnold et al. [21]. In short, these examples illustrate future directions for the modification of pre-existing tips, or even full fabrication on pre-finished SPM cantilevers. For that, FEBID’s additive, direct-write 3D character is the ideal basis for rapid prototyping of specialized AFM tips, which can be integrated in small scale fabrication lines by industry. The unique possibilities justify the higher costs; furthermore, most of the concepts described here remain elusive for standard nanofabrication routes.

### 5.2. Challenges

3D-FEBID is versatile and flexible, but there are limitations. Challenging design elements are truly horizontal segments over a distance of more than 500 nm, as incremental deposition reacts very sensitively to any changes in temperature and/or cross-sectional shape [33]. The fabrication of downward bended segments is even more challenging, although demonstrated in-principle for FIB processing, whose deposition process is similar to FEBID [145]. A workaround for low-angle/descending segments is a substrate pre-tilt, as recently demonstrated by the work-group around Amalio Fernández-Pacheco [146], which requires a very thoughtful upfront design. Alternatively, supporting structures, as typically used in 3D printing technology, can be introduced as well. Depending on the application concept, support structures can be made of non-interfering materials (e.g., SiO_X_ for electrically conductive concepts) or locally removed afterwards via FIB processing [34], however, this can be risky for the main structure.

Most efforts concerning shape-flexibility are currently going into the advancement of process file generators. The main goal of these activities is a software platform, which allows easy and, foremost, reliable transfer of the initial 3D model in a real structure. For that, an intuitive and user-friendly interface is planned, which accepts classical 3D files (e.g., from Blender™ or conventional computer-aided design (CAD) programs) as input data. These activities also include the implementation of closed and semi-closed 3D elements and compensation modules for the highest possible spatial precision. The latter, in particular, considers long and tall architectures, which can suffer from local beam heating, as recently revealed [147] and studied in detail in another article of this special issue by Fowlkes et al. [148].

## 6. Scalability and Speed

In principle, FEBID is a single beam technology, which inherently limits the throughput. Hence, this technology was mostly used for the fabrication of single objects in the past, with exceptions, such as multi-object plasmonic arrays [25,149]. The latter, however, clearly demonstrate FEBID’s scalability, as shown by another example in Figure 1e, which is composed of 640 freestanding single 3D objects across an area of 170 µm^2^ without losing the shape fidelity. Further examples, such as meshed towers, 10 µm high, or complex architectures composed of 1296 single elements (a replica of the Louvre in Paris, France) can be found in reference [44]. While such multi-branch structures can become interesting for optical filters or sensors [150], SPM-relevant tasks typically require a low number of individual elements, however, these need to be fabricated on a higher number of cantilevers. In that context, programmable sample stages in combination with image recognition systems and beam quality control approaches can be a possible route for the automated fabrication of advanced AFM tips on a larger scale. In that context, Jenke et al. demonstrated a FEBID-based, multi-step procedure, which might be applicable for batch fabrication [151]. The authors used FEBID for Au seeding, followed by vapor–liquid–solid (VLS) growth of pure Si nanowires (SiNW), capped with the initial Au cluster. To control the growth direction, a guiding template was fabricated via FIB upfront, leading to straight SiNW with lengths and widths in the micron and sub-100 nm regime. Due to the Au cap, such probes can be used for optical applications, as described in the previous chapter. This approach is particularly interesting, as the FEBID step can be finished in about 10 s, while the subsequent VLS steps can collectively be applied, which reduces the overall fabrication times. While such an approach would fulfill more industrially related demands, the fabrication of individual elements still uses single beams. To overcome that limitation, multi-beam approaches are a possible future scenario. So far, Hagen et al. have demonstrated parallel FEBID processing using 196 electron beams, whose core element, the beam splitter, can be retrofitted in many SEMs and dual-beam microscopes [152,153]. An even more exciting platform was introduced 2016 by IMS nanofabrication, where 262,144 programmable electron beams were used for electron beam lithography for the parallel processing of an area of 82 µm × 82 µm with a resolution of 14 nm [154], while further performance improvements are currently in progress [155]. If such machines could be equipped with proper gas injection systems, FEBID could be leveraged to a large-scale technology for industrial applications.

## 7. Conclusions

The direct-write fabrication of freestanding 3D geometries via focused electron beam-induced deposition (FEBID) has experienced a remarkable evolution from a scientifically oriented method into a versatile 3D-nanoprinting technology. FEBID’s main advantages are the additive, direct-write character, in combination with its 3D capabilities, extremely low demands on substrate material/morphology, (routinely) achievable feature sizes down to 20 nm, material variability, and the high design flexibility. These aspects are highly beneficial for SPM tip modification, or even full tip fabrication on pre-existing tips or pre-finished cantilever platforms, respectively. Due to 3D-FEBID’s design flexibility, even more advanced nano-probe concepts can be explored, which are extremely challenging or even impossible via alternative nano-fabrication approaches. By that, this technology represents an ideal basis for advanced nano-probe fabrication in scanning probe microscopy.
